# Differential Tolerance to *Calonectria pseudonaviculata* of English Boxwood Plants Associated with the Complexity of Culturable Fungal and Bacterial Endophyte Communities

**DOI:** 10.3390/plants10112244

**Published:** 2021-10-21

**Authors:** Ping Kong, Melissa Sharifi, Adria Bordas, Chuanxue Hong

**Affiliations:** 1 Virginia Tech, Hampton Roads Agricultural Research and Extension Center, 1444 Diamond Springs Road, Virginia Beach, VA 23455, USA; chhong2@vt.edu; 2 The Colonial Williamsburg Foundation, P.O. Box 1776, Williamsburg, VA 23185, USA; msharifi@cwf.org; 3 Virginia Cooperative Extension, Fairfax Co., 12011 Government Center Parkway, Fairfax, VA 22035, USA; abordas@vt.edu

**Keywords:** English boxwood, differential tolerance, fungal pathogen, culturable microbiome, endophytic community complexity

## Abstract

Isolated boxwood endophytes have been demonstrated to effectively protect boxwood plants from infection by *Calonectria pseudonaviculata* (*Cps*). However, the roles of endophytes as communities in plant defense are not clear. Here, we demonstrated differential tolerance to *Cps* of English boxwood (*Buxus sempervirens* ‘Suffruticosa’), an iconic landscape plant and generally regarded as highly susceptible, and its link to endophyte complexity. Fifteen boxwood twig samples were collected in triplicates from three historic gardens—Colonial Williamsburg, George Washington’s Mount Vernon and River Farm, and Virginia Tech’s research farm in Virginia Beach in the summer and fall of 2019. A portion of individual samples was inoculated with *Cps* under controlled conditions. Significant differences in disease severity were observed among samples but not between the two seasons. Examining the endophyte cultures of the summer samples revealed that bacterial and fungal abundance was negatively and positively correlated with the disease severity. Nanopore metagenomics analysis on genomic DNA of the tolerant and susceptible group representatives confirmed the associations. Specifically, tolerant English boxwood plants had an endophyte community dominated by *Bacilli* and *Betaproteobacteria*, while susceptible ones had a distinct endophyte community dominated by *Gammaproteobacteria*, *Actinobacteria*, and diverse fungi. These findings may lead to boxwood health management innovations—devising and utilizing cultural practices to manipulate and increase the abundance and performance of beneficial endophytes for enhanced boxwood resistance to *Cps*.

## 1. Introduction

Boxwood blight is a destructive fungal disease [1]. Among the most susceptible cultivars is English boxwood (*Buxus sempervirens* ‘Suffruticosa’), an iconic landscape plant in American and European gardens. The widespread planting of English boxwood, along with interstate and international trade of mass boxwood plant stocks, has presented an overwhelming challenge to blight prevention and control in private and public gardens as well as the ornamental horticulture industry [2,3].

The current boxwood plant protection paradigm relies largely on fungicide treatment, which is not sustainable and, in some cases, not practical. On the other hand, mulching is a common practice that prevents the blight pathogen in infested soil and diseased plant debris from water splash onto the foliage [4]. However, mulching cannot prevent the pathogen from above-ground sources. Another alternative is to use biofungicides. However, none of them is known to be effective [5]. Although several new agents have been demonstrated to protect boxwood from the blight pathogen [5,6,7,8,9], many steps are yet to be taken to move them from lab to field application. These steps include studies on their survival, colonization, mode of actions, field trials, development of successful formulations for field application, and registration regulation [10,11]. Breeding and the use of resistant plant cultivars is a compelling long-term solution [1]. However, even with more resistant cultivars available, rapid replacement of English boxwood may be difficult due to economic, historical, and sentimental reasons. Preservation of this historical cultivar has been a great challenge.

Bacterial and fungal endophytes as important microbial resources for sustainable agro-food system have drawn much attention in recent years because of their roles in plant health and defense against pathogens [12,13,14]. However, most existing research has focused on individual isolates that trigger the first layer of plant defense [6,8,12,15,16,17,18,19]. Endophytes working inside the plant as communities that affect plant resistance or that work as the second layer of defense [17] has been poorly understood until recently. Carrión et al. [20] showed that once the pathogen bypasses this first barrier and establishes physical interaction with the host cortical cells, the innate mechanisms of plant defense and the endophytic microbiome response are triggered. The latter is enriched with *Chitinophagaceae* and *Flavobacteriaceae,* producing pathogen-suppressive molecules such as chitinases, nonribosomal peptide synthetases (NRPSs), and polyketide synthases (PKSs). These findings demonstrate that plant resistance depends not only on the genetic background but also on association with beneficial microbial taxa at both layers of protection. Endophytes play an important role in expanding the plant’s genomic and metabolic capabilities [17].

This study used English boxwood to exclude the genetic impact and investigated the impact of endophytes as communities on plant tolerance. We evaluated plant tolerance to *Cps* under a controlled environment, examined their culturable fungal and bacterial endophytes, and performed metagenomic sequencing on endophyte cultures of selected plant samples with different levels of tolerance to *Cps*. We demonstrated that the differential tolerance of individual plants from the same cultivar under the same high disease pressure was associated with the complexity (the membership and the abundance of each member) of their culturable endophyte communities. Plant susceptibility is linked to the complexity of fungal endophytes, while plant tolerance is related to the complexity of some bacterial endophytes. This study provides insights into the role of endophytes in the differential plant tolerance beyond genetics and the potential applications of the endophytes in safeguarding historical boxwood plantings.

## 2. Results

### 2.1. Blight Tolerance Varied among English Boxwood Plants Sampled

**Figure 1:**
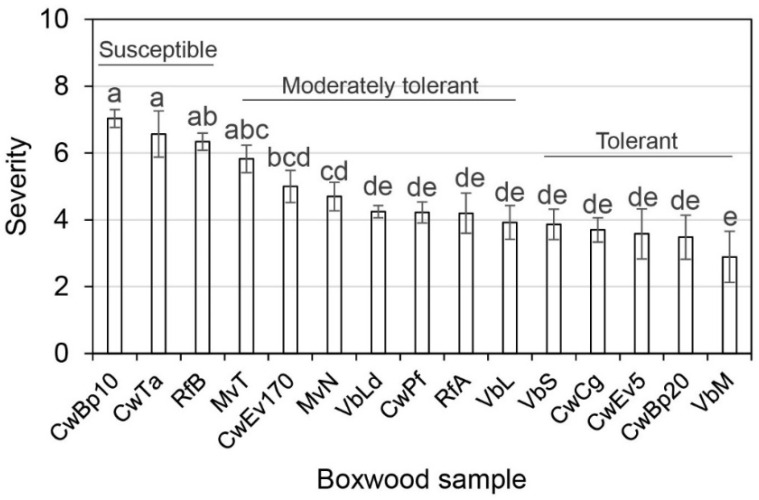
Differential blight tolerance of English boxwood twigs collected from three historic gardens in Virginia—Colonial Williamsburg (Cw), George Washington’s Mount Vernon (Mv) and River Farm (Rf), and the Virginia Tech’s research farm in Virginia Beach (Vb). Each column represents the mean disease severity topped by a standard error bar (*n* = 6). Columns topped with a shared letter do not differ according to the least significant difference test at *p* = 0.05.

Fifteen English boxwood twig samples were collected from three historic gardens—Colonial Williamsburg (Cw), Geroge Washington’s Mount Vernon (Mv), River Farm (Rf), and Virginia Tech’s research farm in Virginia Beach (Vb), VA, in both the summer and fall of 2019 (Table S1). The sample twigs were evaluated for their tolerance to the boxwood blight pathogen, *Calonectria pseudonaviculata* (*Cps*). Although English boxwood is generally considered highly susceptible to *Cps*, blight severity varied among the plants sampled (Figure 1). Significant variations were present among the plant samples (*p* < 0.0001) but not among the sample replicates (*p* = 0.8888) and seasons (*p* = 0.6974). All plant samples had identical genetic makeup and were evaluated under the same conditions. These variations were hypothesized to be attributed to their endophytic and epiphytic microbiome and/or epigenetics.

### 2.2. Endophyte Culture Complexity and Its Blight Tolerance Relation

**Figure 2:**
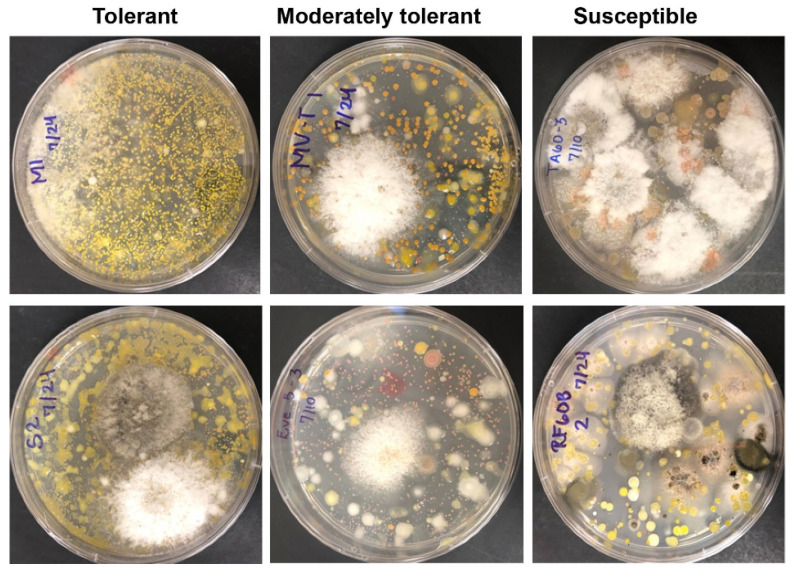
Diverse bacterial and fungal endophytes on potato dextrose agar media spread with the preparation of boxwood leaves with different levels of blight tolerance and incubation at 23 °C. Pictures were taken 10 and 14 days after the inoculation.

The microbial cultures in Petri dishes spread with leaf preparation of individual samples showed two distinct patterns. Those from tolerant and moderately tolerant plants appeared to have more bacterial than fungal colonies, while those from susceptible plants had the opposite ratio (Figure 2).

**Figure 3:**
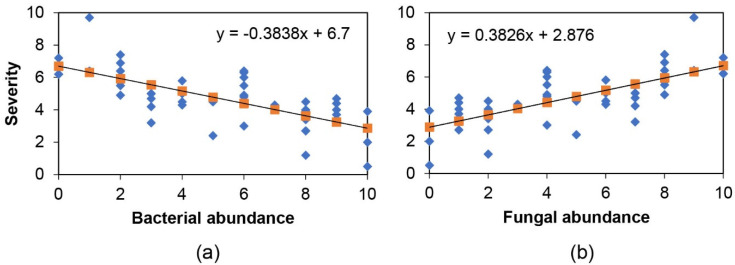
Correlation of boxwood blight severity with bacterial (**a**) and fungal (**b**) abundance in the summer sample culture dishes. Blue diamonds are the samples at the corresponding abundance level. Orange squares are points connecting the trend line.

The summer samples’ bacterial and fungal abundance rating and colony type count confirmed the observations on the culture dishes (Table S2). The highest coefficients were present between the blight severity and the abundance of the bacterial communities, while there was no correlation between the severity and fungal colony type count (Table 1). Further regression analyses indicated that blight severity decreased with increasing bacterial abundance (Figure 3a). Contrarily, disease severity increased with increasing fungal abundance (Figure 3b).

### 2.3. Differential Complexity of Endophytic Bacterial and Fungal Communities between Tolerant and Susceptible Plants

Two plant samples, VbM and RfB, were selected to represent tolerant and susceptible plant groups, respectively. Using Nanopore metagenomic sequencing, three replicate sample endophytic cultures of the representatives were analyzed for the diversity of bacterial and fungal communities. The genomic DNA of the endophyte cultures generated QC-pass sequence reads ranging from 20,621 to 59,437 per sample. Comparing WIMP NCBI-produced taxonomy trees at four taxonomic ranks revealed substantial differences in the endophyte complexity (composition and abundance) between tolerant and susceptible samples.

**Figure 4:**
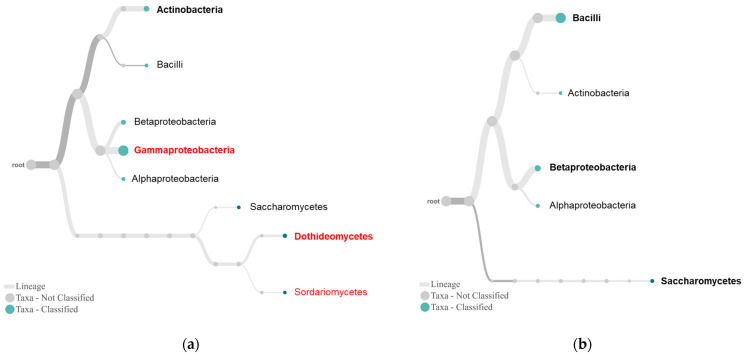
Endophytes in susceptible RfB (**a**) and tolerant VbM (**b**) plants. By default, the trees were constructed with WIMP presenting the top 30 taxa at abundance >1% and a taxonomic rank of class. Taxonomic lineages in bold are predominant in the samples, and those in color are unique to the plant group.

The phylum tree (Figure S1) showed that bacterial *Cyanobacteria* was unique; *Firmicutes* was dominant in the tolerant plants, while *Actinobacteria* and fungal *Ascomycota* were dominant in susceptible plants. The class trees (Figure 4) showed further differences in the bacterial and fungal communities between the tolerant and susceptible plants. First, *Gammaproteobacteria* and fungal *Dothideomycetes* and *Sordariomycetes* were unique, and *Actinobacteria* was predominant in the susceptible plant (Figure 4a). In contrast, *Actinobacteria* was subservient while *Bacilli*, *Betaproteobacteria*, and fungal *Saccharomycetes* were more dominant in the tolerant plant, although there were no unique lineages (Figure 4b). In particular, the number of fungal classes appeared to increase with increasing plant susceptibility levels, as indicated when the trees of individual samples were compared (Figure S2).

**Figure 5:**
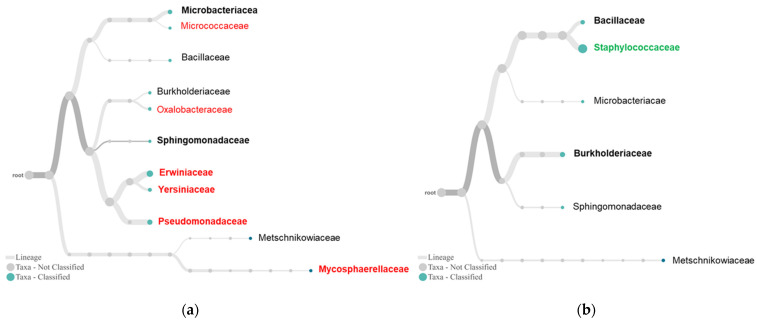
Endophytes in susceptible RfB (**a**) and tolerant VbM (**b**) plants. By default, the trees were constructed with WIMP presenting the top 30 taxa at abundance >1% and a taxonomic rank of family. Taxonomic lineages in bold are predominant in the samples, and those in color are unique to theplant group.

Similar divergence was observed between tolerant and susceptible plants in the family tree (Figure 5). The susceptible plants had six unique families with three predominant bacterial families and one fungal family (Figure 5a). Comparatively, the tolerant plants had one unique bacterial family, *Staphylococcaceae*, and two predominant ones, *Burkholderiaceae* and *Bacillaceae* (Figure 5b).

**Figure 6:**
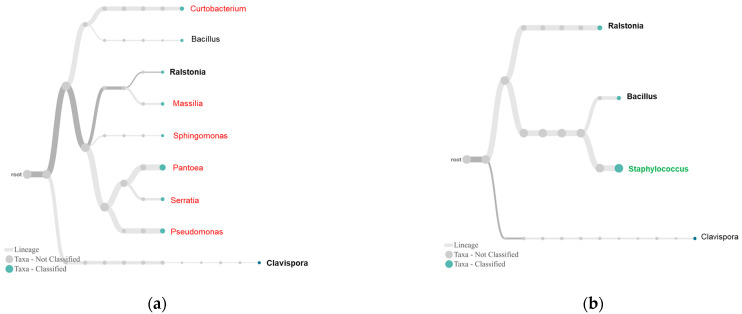
Endophytes in susceptible RfB (**a**) and tolerant VbM (**b**) plants. By default, the trees were constructed with WIMP presenting the top 30 taxa at abundance >1% and a taxonomic rank of genus. Taxonomic lineages in bold are predominant in the samples, and those in color are unique to the plant group.

In the genus trees (Figure 6), although tolerant and susceptible plants shared the only genus *Clavispora*, the susceptible plants were more dominated by the genus (Figure 6a) than the tolerant plant (Figure 6b). For bacterial endophytes, there were six unique genera in the susceptible plants. Among these genera, *Pantoea*, *Serratia*, and *Pseudomonas* were predominant (Figure 6a). The number of bacterial genera in the tolerant plants was fewer than that in the susceptible plant, and the community was dominated by *Staphylococcus*, which was not seen in the susceptible plants. Other important members in this community included *Ralstonia* and *Bacillus* (Figure 6b).

## 3. Discussion

This study demonstrated that differential disease tolerance of English boxwood plants under the same high disease pressure is associated with the complexity of their culturable endophyte communities. The role of endophyte communities as the second layer of plant defense was recently reported in roots [20]. This study has extended this role to plant foliage. This extension has several implications.

English boxwood, an iconic landscape plant in American and European gardens [21], is regarded as highly susceptible to *Cps* and is commonly used as a positive control when evaluating boxwood [22,23,24] and sweet box [25] for their blight resistance [1]. Protecting this landmark plant from *Cps* has presented a significant challenge for homeowners and public/historic garden managers. The fact that plants of this cultivar have differential blight disease tolerance related to endophyte communities sheds light on safeguarding and preserving such an endangered iconic plant. Plant inoculation with isolated endophytes has been demonstrated temporarily effective in the disease mitigation [6,8] and may also lead to the first layer of plant defense like other biocontrol agents or plant–rhizosphere microbiomes [9,12,15,16,17,18,19]. On the other hand, microbial community coalescence has allowed the encounter and interaction of the entire microbial communities [26]. With this concept, methods such as cutting or grafting propagation with tolerant plants may be used for endophytic microbial community transfer between the mother plants and their offspring, allowing the second layer defense in play for permanent tolerance. Alternatively, preserving historic boxwood plantings may be achieved by devising and utilizing cultural practices to enhance the abundance and performance of culturable beneficial bacterial endophyte groups identified in this study.

Several lines of evidence support that the differential tolerance of individual English boxwood plants sampled and evaluated in this study is closely related to the complexity of culturable bacterial and fungal endophyte communities. At the culture level, plant tolerance to *Cps* increased with increasing bacterial endophyte colony types and abundance while decreasing with increasing fungal abundance (Table 1, Figure 1, Figure 2 and Figure 3). Metagenomic DNA sequencing of the cultured endophyte communities confirmed these associations.

The sequencing also provided additional insights into these associations (Figure 4, Figure 5 and Figure 6). For fungal endophytes, *Saccharomycetes* presented as a single dominant class in the tolerant plants. As budding yeasts, they live as decomposers, feeding on dead and decaying wood, leaves, litter, and other organic matter. Some endophytic yeasts, such as species in the genus *Rhodotorula*, have been reported as biocontrol agents [27]. *Clavispora* was the only genus detected in both tolerant and susceptible plants (Figure 6). The active species of this genus and other genera involving boxwood tolerance remain unclear. Regarding bacterial endophytes for plant tolerance, *Betaproteobacteria* and *Bacilli*, two classes with the most known biological control agents, are more diverse and abundant in the tolerant than susceptible plants (Figure 5). *Burkholderiaceae* is the most dominant subgroup of *Betaproteobacteria* in the tolerant plants (Figure 5). We recently isolated *Burkholderia* sp. SSG that was involved in the disease symptom reversion from infected boxwood leaves by *Cps*. The isolate is highly effective against boxwood blight [6] and many other plant diseases caused by oomycetes, bacterial and viral pathogens [18]. Its role in plant defense has been attributed to its strong capacity to produce antibiotics like root pathogen suppressive endophytes [20,28] and plant growth-promoting compounds [29]. *Bacillaceae* is the second most dominant subgroup of *Bacilli* in the tolerant plant (Figure 5). *Bacilli* produce lipopeptides, one of the most important classes of antimicrobial compounds [30,31]. A *Bacillus* strain has been isolated from tolerant boxwood leaf endophyte cultures, which is *Cps*-suppressive (Kong, unpublished data). Another subgroup in *Bacilli* is *Staphylococcaceae* or *Staphylococcus* at family and genus levels, which is unique in the tolerant plants (Figure 5 and Figure 6). Information about this group as biocontrol agents remains unknown. Nevertheless, this study highlights the significance of endophytic *Burkholderia* and *Bacillus* species in boxwood tolerance to *Cps*.

The NCBI taxonomy trees produced by the Nanopore EP2ME WIMP may lead to identifying plant tolerance-associated endophyte lineages and their abundance in sample endophyte cultures. The trees at all four taxa ranks can somehow differentiate tolerant from susceptible plants, although the class tree provides the best resolution, particularly in fungal lineages that differentiate tolerance between tolerant and susceptible plants (Figure 4) and tolerance levels in these plants (Figure S2). A genus tree with less resolution specifically for fungi was unexpected, which may be caused by the limited sequencing depth or available reference databases used by WIMP. Barcoding amplicons of specific DNA fragments for fungi and bacteria may facilitate sequencing depth, improving genus tree’s resolution.

Among the three historic gardens sampled in this study, Colonial Williamsburg has the most extensive boxwood plantings with almost exclusively ‘Suffruticosa’ of different ages from 5 to 170 years. Comparatively, the two other gardens were limited in plantings with all 70 years and older. While disease severity variations among the plants sampled in this study were observed, their association with plant age was unclear. It was not possible to determine such association because of the differences in plant availability among the three gardens, nor was this the objective of this study. This study focused on differential disease tolerances of sampled plants and their bacterial and fungal endophyte communities. For metagenomics analysis, the selected sample plants: VbM and RfB were 10 and 100 years old, respectively, at the time of sample collection. How age may affect boxwood susceptibility to *Cps* and how the boxwood endophyte communities may change with plant age are important questions for future investigation.

## 4. Materials and Methods

### 4.1. Plant Samples

English boxwood (*Buxus sempervirens* ‘Suffruticosa’), an iconic landscape plant highly susceptible to *Calonectria pseudonaviculata* (*Cps*), was selected as the cultivar of study; three historic gardens—Colonial Williamsburg in Eastern Virginia, George Washington’s Mount Vernon and River Farm in Northern Virginia, along with Virginia Tech’s research farm in Virginia Beach, VA, USA—as the sites for sampling. At each site, at least two samples were taken from different plants with three replicates each (Table S1). Sampling was done twice in the summer and fall of 2019, respectively. Each replicate sample included ten 10-cm long twigs. All sample twigs were stored at 4 °C and processed within 1–2 days of sampling.

### 4.2. Evaluation of Boxwood Tolerance to Cps

Three twigs were randomly selected from each plant sample replicate and inserted into prewet Gro-Block™ plugs (Grodan, CA, USA) with 10% Hoagland’s solution to keep the material alive. For inoculation, the twigs were placed in UltraTM Latching Storage Boxes (66 cm × 41 cm × 50 cm, Sterilite Corporation, Townsend, MA, USA) using a randomized complete block design. A *Cps* isolate, 12A0 collected from Virginia, was used for inoculation. The inoculum was prepared as described previously [6]. Inoculation was done by block with a hand sprayer. Each twig received about 7 mL of inoculum at 3 × 10^4^ conidia mL^−1^. Inoculated twigs were kept in closed storage boxes with a small amount of water in the bottom for 48 h to create a moist environment and facilitate infection. After that, box covers were removed, and the plants were misted every other day to maintain leaf wetness.

Blight severity was rated at 5- and 10-days post-inoculation (dpi), respectively, on a 0–10 scale: 0 = no disease, 1 = 1–10%, 2 = 11–20%, …, and 10 = 91–100% leaves diseased. Plant samples with mean disease severity of 0–3.9 were considered tolerant (T), and those of larger than 6.0 were as susceptible (S), while those in-between were moderately tolerant (M).

### 4.3. Endophyte Culture Preparation from Samples

The same sets of samples used for Section 4.2 were used here. For each replicate sample, one leaf was excised from each of the three twigs and pooled before inoculation. The pooled leaves were surface-sterilized with 70% ethanol and 10% bleach, respectively, followed by three rinses in a large volume of sterilized distilled water (SDW), then blotted dry with a sterilized paper towel to remove epiphytes and associated DNA residues. They were then cut into small pieces and placed in a presterilized 2 mL microtube containing 1 mL SDW, 0.3 mL 1.4 mm Zirconium beads (MP Biomedicals, Santa Ana, CA, USA), and one ¼″ (6.35 mm) ceramic sphere (OPS Diagnostics, Lebanon, NJ, USA). Leaf pieces in the tubes were homogenized for 3 × 30 s at speed 6 on FastPrep-24™ Classic bead beating grinder and lysis system (MP Biomedicals, Santa Ana, CA, USA). An 0.3-mL aliquot of the resultant suspension was plated on potato dextrose agar (PDA, Sigma-Aldrich, St. Louis, MO, USA) in each 9-cm replicate Petri dish. These dishes were incubated at 23 °C for 7–10 days.

### 4.4. Evaluation of Cultured Endophyte Complexity

The culture dishes were evaluated for colony type count and abundance. Bacterial and fungal abundance in the dishes was rated, separately, using a 0–10 scale: 0 = no colony, 1 = colonies occupy 1–10% of the dish, 2 = 11–20%, …, and 10 = 91–100%. Bacterial and fungal colony types in each dish were enumerated separately based on the colors and sizes.

### 4.5. Endophyte Culture DNA Extraction and Nanopore Sequencing and Analyses

Two samples were selected, with one representing the tolerant group and the other for the susceptible group. Accordingly, three culture dishes spread with leaf preparation of each selected sample were used for metagenomic analysis. Briefly, all colonies in each dish were harvested and extracted for DNA using NucleoSpin^®^ Microbial DNA (Takara Bio, Mountain View, CA, USA). Extracted DNA was quantified for concentration with Quantus™ Fluorometer (Promega Corporation, Madison, WI, USA) and measured for OD 260/280 with Du 800 UV/VIS spectrophotometer (Beckman Coulter, Pasadena, CA, USA)

A genomic sequencing library was prepared for three replicates of selected tolerant and susceptible plant endophyte culture DNA samples by following the rapid barcoding protocol for SQK-RBK004 Kit of Oxford Nanopore Technologies (ONT, Cambridge, UK). A total of 400 ng of DNA of each sample with OD 260/280 ≥ 1.8 was used for barcoding with RB1-6. After barcoding and clean-up, the equimolar of each barcoded sample were pooled for rapid adapter ligation. Sequencing was run on the MinION sequencer (ONT, Cambridge, UK). Sequencing and base calling were performed through MinKnow for windows (version 19.06.7) using a fast base calling model at a quality score (QS) 10 targeting accuracy of over 90%.

The basecalled reads or FASTQ files were analyzed with WIMP (What’s In My Pot) workflow (rev.2020.05.19) on the EP2ME platform in the Desktop Agent (ONT, Cambridge, UK). WIMP uses the Centrifuge software to classify reads to taxon ID based on the NCBI taxonomy reference database. The output report was used to present the endophytic bacterial and fungal diversity in respective samples.

### 4.6. Data Analysis and Statistics

Plant tolerance evaluation data were subjected to a homogeneity test and subsequently pooled for further analyses. Analysis of variance was conducted using the Statistical Analysis Software Version 9.4 (SAS Institute, Cary, NC, USA). Disease severity means were separated by plant according to the least significant difference at *P* = 0.05. The correlation between blight tolerance and the number of colony types and their significance was determined using Excel Data analysis’s regression function.

NCBI Taxonomy trees for tolerant or susceptible sample representative were constructed with WIMP output reports by default; each tree represents the top 30 taxa of a sample reads at minimum abundance cutoff *n* = 1%. Taxa were presented at a rank of phylum, class, family, or genus depending on the separation between resistant and susceptible samples.

## Figures and Tables

**Table 1:** Correlation between blight severity and endophyte culture complexity.

Community	Abundance		Colony Type	
	*p*-Value	R^2^	*p*-Value	R^2^
Bacteria	<0.0001	0.4752	<0.0001	0.3755
Fungi	<0.0001	0.4796	0.2806	0.0270

## Data Availability

The metagenomic sequences of the cultural endophytes from six representative resistant and susceptible plant samples have been deposited at DDBJ/ENA/GenBank under BioProject PRJNA755950, accession: SRX11824923-28; SRR15526207-202, BioSample accession SAMN20847028-33.

## Supplementary Materials

The following are available online at https://www.mdpi.com/article/10.3390/plants10112244/s1, Table S1: Blight severity of English boxwood samples; Table S2: Complexity of bacterial and fungal endophytes of plant samples in summer 2019 according to the culture morphology on potato dextrose agar media; Figure S1: Endophytes in susceptible RfB (a) and tolerant VbM (b) plants. By default, the trees were constructed with WIMP presenting the top 30 taxa at abundance >1% and a taxonomic rank of phylum; Figure S2: Endophytes in susceptible replicates (RfB 1, 2, 3) with blight severity 7.4, 6.9, and 6.0 and tolerant replicates (VbM 1, 2, 3) with blight severity 2.4, 1.2, and 0.5 of English boxwood plants. The class trees were constructed with WIMP by default, presenting the top 30 taxa at abundance >1%.
