# Supplementary figures and images for: Differential Tolerance to Calonectria pseudonaviculata of English Boxwood Plants Associated with the Complexity of Culturable Fungal and Bacterial Endophyte Communities
Source: Plants (Basel). 2021 Oct 21;10(11):2244. doi: 10.3390/plants10112244 (PMC8619141; doi:10.3390/plants10112244)

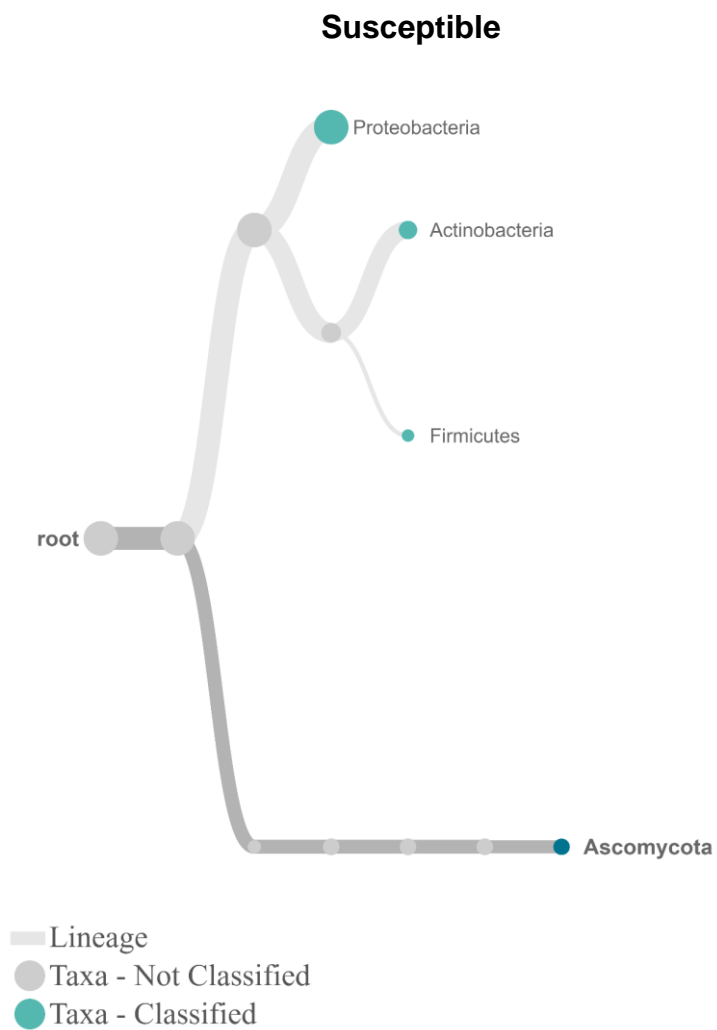

(a)

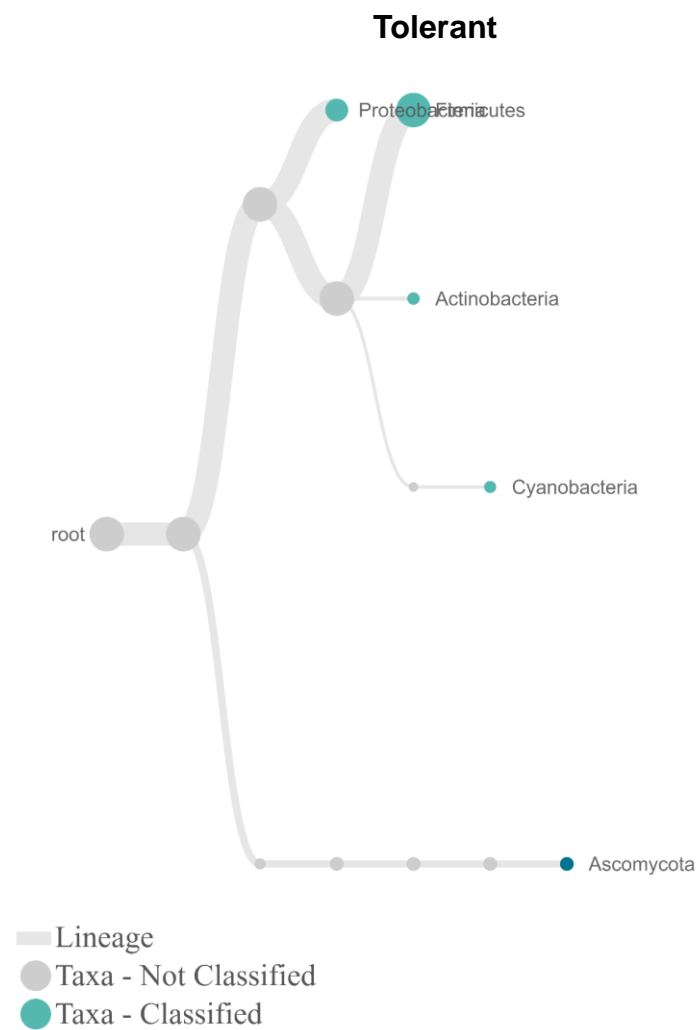

(b)

Supplement: Supplementary file 1 [file plants-10-02244-s001.zip › FigS1-phylum-ppt.pdf]

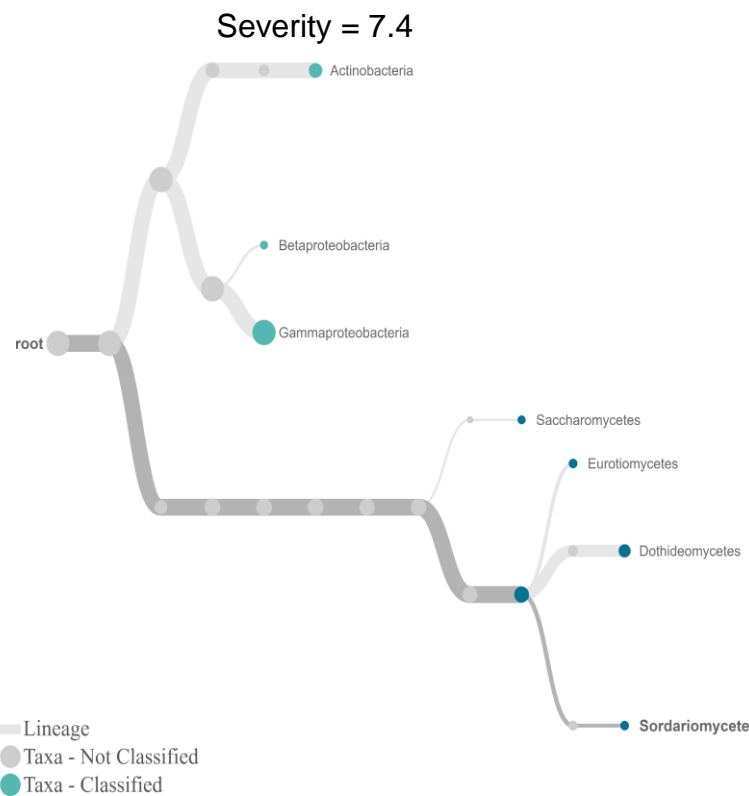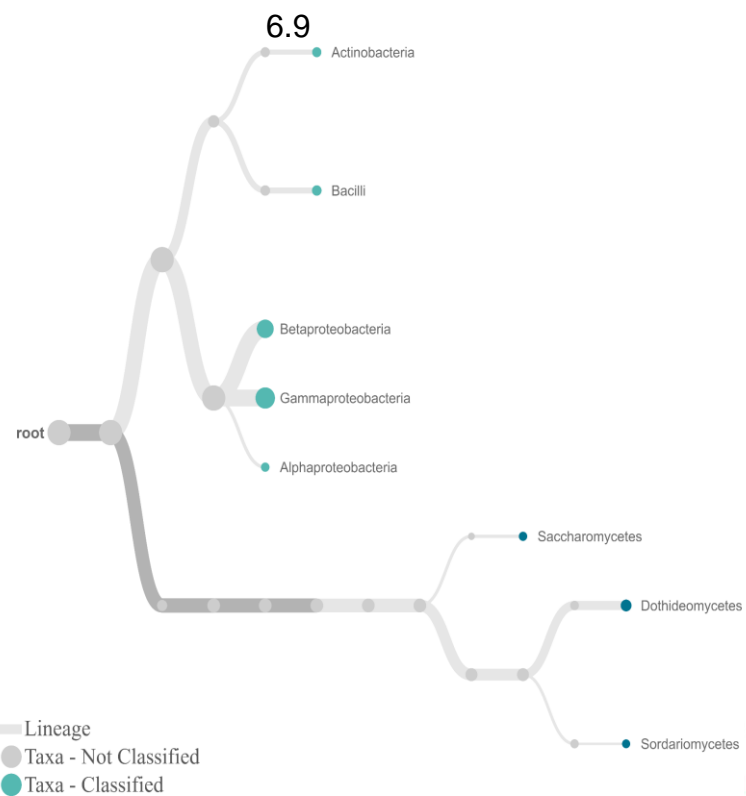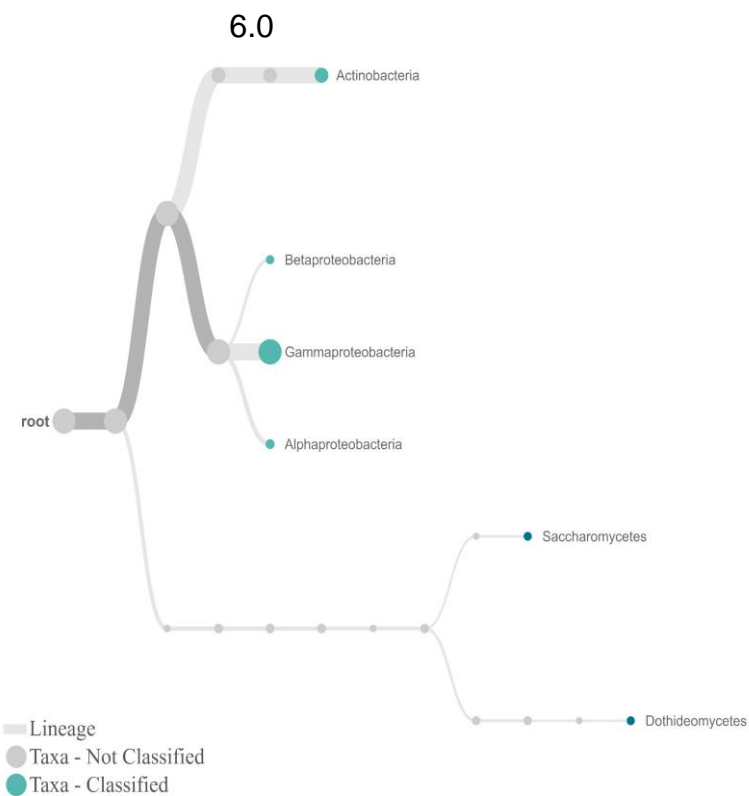

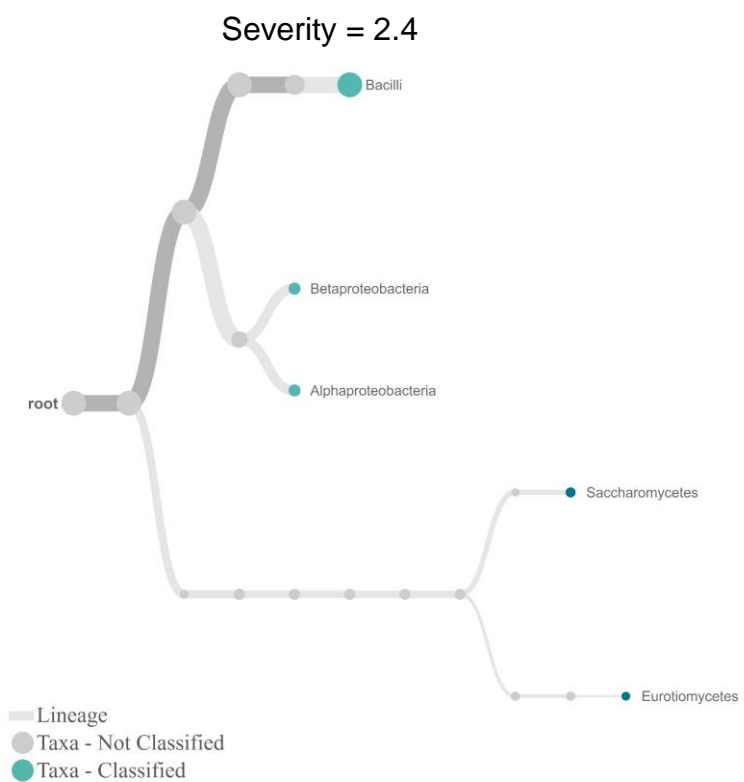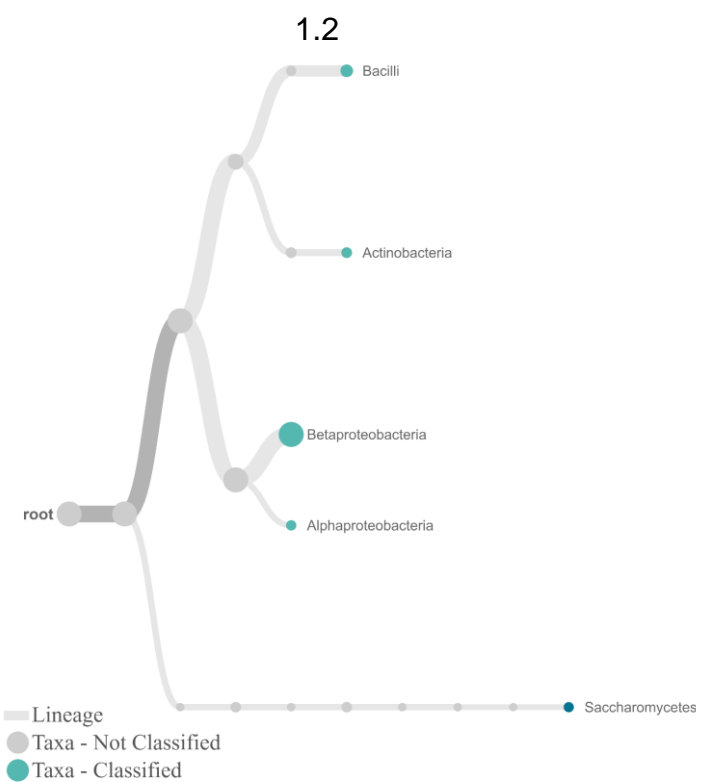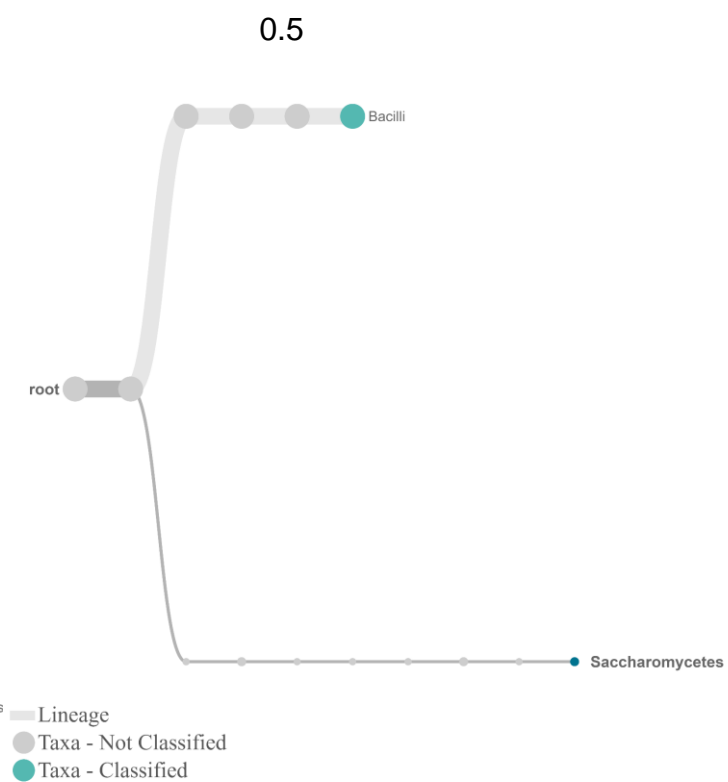

Supplement: Supplementary file 1 [file plants-10-02244-s001.zip › figS2-replicates-class.pdf]
